# Everyday Uses of Music Listening and Music Technologies by Caregivers and People With Dementia: Survey and Focus Group Study

**DOI:** 10.2196/54186

**Published:** 2024-08-27

**Authors:** Dianna Vidas, Romina Carrasco, Ryan M Kelly, Jenny Waycott, Jeanette Tamplin, Kate McMahon, Libby M Flynn, Phoebe A Stretton-Smith, Tanara Vieira Sousa, Felicity A Baker

**Affiliations:** 1 School of Computing and Information Systems The University of Melbourne Parkville Australia; 2 Facultad de Comunicación y Artes Audiovisuales University of Las Américas Ecuador Quito Ecuador; 3 School of Computing Technologies RMIT University Melbourne Australia; 4 Faculty of Fine Arts and Music The University of Melbourne Melbourne Australia; 5 Centre for Research in Music and Health Norwegian Academy of Music Oslo Norway

**Keywords:** dementia, dementia care, technology, music technology, mobile phone

## Abstract

**Background:**

Music has long been identified as a nonpharmacological tool that can provide benefits for people with dementia, and there is considerable interest in designing technologies to support the use of music in dementia care. However, to ensure that music technologies are appropriately designed for supporting caregivers and people living with dementia, there remains a need to better understand how music is currently used in everyday dementia care at home.

**Objective:**

This study aims to understand how people living with dementia and their caregivers use music and music technologies in everyday caring, as well as the challenges they experience using music and technology.

**Methods:**

This study used a mixed methods design. First, a survey was administered to 13 people living with dementia and 64 caregivers to understand their use of music and technology. Subsequently, 18 survey respondents (family caregivers: n=12, 67%; people living with dementia: n=6, 33%) participated in focus groups regarding their experiences of using music and technology in care. Interview transcripts were analyzed using reflexive thematic analysis.

**Results:**

Most of the survey respondents (people living with dementia: 9/13, 69%; family caregivers: 47/63, 75%) reported using music *often* or *very often* in their daily lives. Participants reported a range of technologies used for listening to music, such as CDs, radio, and streaming services. Focus groups highlighted the benefits and challenges of using music and music technologies in everyday care. Participants identified using music and music technologies to regulate mood, provide joy, facilitate social interaction and connection, encourage reminiscence, provide continuity of music use before and after the dementia diagnosis, and make caregiving easier. The challenges of using music technology in everyday caring included difficulties with staying up to date with evolving technology and low self-efficacy with technology for people living with dementia.

**Conclusions:**

This study shows that people with a dementia diagnosis and their caregivers already use music and music technologies to support their everyday care needs. The results suggest opportunities to design technologies that enable easier access to music and to support people living with dementia with recreational and therapeutic music listening as well as music-based activities.

## Introduction

### Background

Worldwide, >55 million people are currently living with dementia, making dementia one of the leading causes of disability among older adults globally [1]. While many people living with dementia are aged ≥65 years, younger onset dementia can affect people of different ages. As well as difficulties with cognition, people living with dementia may experience a range of behavioral and psychological symptoms, such as agitation, anxiety, irritability, and depression [2]. These difficulties place considerable demands on family and other “informal” caregivers, who provide the majority of care for people with dementia living in the community [3]. Although informal caregivers frequently report that they need additional support and information [4], they are often reluctant, or lack knowledge, to fully use services [5], leaving many of them feeling burdened and experiencing depressive symptoms [6]. As a result, caregivers and people with dementia need accessible strategies to support their everyday care needs [7-10].

Music is a nonpharmacological tool that can provide numerous benefits for people with dementia [11-14]. Music therapy, where music is used as an intervention by a trained therapist, has long been shown to assist in managing symptoms of dementia, such as agitation and depression, and can support interpersonal connection between people with dementia and their caregivers [11,15-19]. While music therapy is a promising avenue for improving the lives of people with dementia and their caregivers, it may not be readily accessible for all people with dementia, particularly those who are less able to participate in activities outside of the home [20]. Caregiver training programs informed by music therapy, where caregivers are trained by music therapists in intentional uses of music, have received attention as a method of providing quality informal care [21]. While experiences informed by music therapy can strategically target specific care needs for people living with dementia, music activities not informed by therapeutic approaches can still be valuable for a range of purposes across the continuum from therapeutic to recreational experiences [11].

In the absence of a music therapist, recreational music experiences such as music listening or singing can also provide important everyday support for improving quality of life [11,22]. In the aged care setting, recreational music listening can provide people with dementia with enjoyment, relaxation, social connectedness, entertainment, and mood regulation [13,23]. Similar benefits of music use have been identified for older adults with dementia living at home, with music reducing agitation, improving cognition, and enhancing social well-being and connection [19,24]. However, much of the research on this topic has focused on identifying benefits gained from participating in organized music activities, such as community singing groups [18]. There has been less research investigating how informal caregivers use music and music technologies in everyday home-based care [25]. One exception, a study by Elliott et al [24], used a case study approach to examine the musical experiences of 3 people living with dementia and their caregivers, but it did not examine the technologies used to access music. As such, there remains a need for further research to understand the role of music in everyday dementia care as well as the role of technology in facilitating access to music for people with dementia and their caregivers.

While technology can support both therapeutic and recreational uses of music in dementia care, existing research has focused primarily on using technology to support recreational uses of music, primarily in formal care settings. Several technologies have been developed and trialed to support music listening and music-based activities for people living with dementia [26,27]. Researchers have designed and trialed technologies that aim to support music for dementia care by bringing enjoyment and social connection to people with dementia through both music and sound [28,29]; for example, Hodge et al [30] created a virtual reality environment of a concert experience, tailored for the specific interests of 1 person living with dementia.

Music-based technologies in the aged care context have been explored as a way to promote an enjoyable, active form of engagement with music that may facilitate connection and shared movement [31-33]. In another example, researchers created a musical interface that aimed to facilitate collaborative music making to foster partnership between aged care residents and professional caregivers [29]. Interactive approaches to designing for musical interaction for people living with dementia extend to mobile apps, which can facilitate collaborative music making [34]. In addition, music therapists use a range of technologies to engage their clients in communication, connection, and music making [35]. This suggests that music technologies have utility for recreational and therapeutic music experiences for people living with dementia; yet, their use by informal caregivers remains understudied. There are substantial differences between formal and informal care settings. Formal care often involves group activities, facilitated by trained caregivers. Conversely, informal care typically takes place in the private home, with family caregivers who may not have training in using specific care strategies in their care. While benefits shown in formal care settings likely apply across contexts, informal caregivers’ existing everyday care practices with mainstream technologies, without training or formal intervention, remain unclear. Furthermore, understanding everyday uses of music technologies in caregiving can help to identify what works well and where opportunities may lie for technology innovation.

### Objectives

In this paper, we aim to understand the role of music and music technologies in the everyday lives of people with dementia and their family caregivers. Here, we define music technologies broadly as technologies used to facilitate listening to (eg, music streaming services and CDs) or playing (eg, a piano app) music. We consider the potential of music technologies both for recreation and for managing the behavioral and psychological symptoms of dementia. Music is now often accessed through technologies such as streaming services, but there may be barriers to using these technologies in the complex environment of family-based dementia care. To create new technologies that support the use of music for people living with dementia at home, we must understand how music technologies are currently used in dementia care and what challenges these groups face in using music with current technologies.

This study used a mixed methods design with 2 phases. An initial survey with family caregivers and people with dementia aimed to investigate their use of music and technology. Survey participants were then invited to take part in a focus group study, which further discussed current practices with music and music technologies in dementia care. We aimed to understand how people with dementia and their family caregivers use music and music technologies as part of their everyday care practices. We further aimed to explore the perceived benefits of using music and music technologies, as well as the perceived challenges that caregivers and people living with dementia face in accessing music through technology.

## Methods

### Ethical Considerations

All procedures were approved by the University of Melbourne Human Research Ethics Committee (2020-14155-13119-4). A plain language statement and consent form were shown at the beginning of the survey, and respondents had to provide consent before proceeding with the survey. Survey respondents could also indicate their interest in participating in focus groups for the main study. Participants who completed the survey were entered into a draw to win an iPad Mini. Survey respondents who were willing to participate in the focus groups were sent an electronic plain language statement and consent form indicating that they could be contacted via phone or email. Participants who provided consent were then contacted by a member of the research team who invited them to take part in web-based focus group discussions. During this contact, the researcher provided an overview of the study and an opportunity for participants to ask questions. Participants could attend the sessions alone or as a dyad (caregiver and person living with dementia attending together). All participants were offered a voucher worth Aus $30 (approximately US $23) for their time.

### Participant Recruitment

#### Survey

An anonymous web-based survey was distributed through Facebook advertisements and StepUp for Dementia Research (a web-based platform that connects volunteer research participants with dementia researchers in Australia) [36], calling for responses from Australian caregivers and people living with dementia. There were no strict eligibility criteria, and participants could self-identify as a person living with dementia or a caregiver.

#### Focus Groups

The focus groups did have eligibility criteria: participants were asked to self-identify that they were literate, able to speak English, and had access to stable internet as well as a laptop or tablet computer to participate in the web-based sessions via Zoom (Zoom Video Communications, Inc).

### Procedure

#### Survey

Study data were collected and managed with REDCap (Research Electronic Data Capture; Vanderbilt University), a web-based platform that supports data capture for research studies [37,38]. After the participants provided consent, the survey asked them for demographic information (age, gender, and level of education). Next, participants were asked about how they currently use music in their daily life, whether music has been an important or meaningful part of their life in the past, their musical preferences, and the technologies used to listen to music. Participants were asked about their experience with digital technologies, such as computers, smartphones, music apps, and dementia-related apps. Finally, participants were asked about their openness to using mobile apps to support daily activities and the ways in which a music-based mobile app might be able to support daily activities. For some questions, caregivers answered both for themselves and the person they cared for, while people with dementia responded for themselves and their caregiver, if relevant. The survey (Multimedia Appendix 1) took approximately 15 minutes to complete.

#### Focus Groups

Five focus group sessions were organized, and participants could choose which session suited them. Each participant attended 1 focus group. All sessions were conducted via Zoom to enable the remote participation of people in different regions of Australia and to mitigate COVID-19 infection risk. Of the 18 participants, only 1 (6%; a caregiver) attended the first focus group, as the other registered participants withdrew from the study at the last minute due to unforeseen circumstances. The second and third focus groups were conducted with caregivers only. The fourth focus group was conducted with caregivers and 1 dyad (a person with dementia and their caregiver), and the fifth focus group was conducted with a mix of dyads and people living with dementia who attended the session independently (without a caregiver). The focus groups, facilitated by 2 members of the research team, were led by a researcher in human-computer interaction, who was supported by a music therapist.

The focus groups followed a protocol divided into 4 phases (Multimedia Appendix 1). Each focus group started with an introduction (5-10 min), with participants and researchers introducing themselves. The second phase for caregivers involved discussing their role as caregivers, their experiences of using music and technology in care, the types of music technologies currently used, and how they believed digital technologies may support care. People living with dementia were similarly asked about their experiences with music and how they use technology to listen to music. The third phase involved discussing how potential future music technologies could respond to their needs, leading into a wrap-up and conclusion (phase 4). The questions were developed by the research team to align with the broader goals of the project, which aimed to understand what caregivers and people with dementia wanted from a mobile music therapy–informed app. The focus groups ranged from 46 to 71 minutes in duration. All focus group sessions were video and audio recorded.

### Data Analysis

Survey responses were analyzed using descriptive statistics; we tabulated frequency data for responses to each question using Stata SE (version 16.1; StataCorp LLC). The audio recordings of the focus groups were transcribed by the researchers. We analyzed the transcripts using reflexive thematic analysis [39,40]. Consistent with the assumptions of thematic analysis, we adopted a constructionist approach, meaning that our interpretation considered the socially constructed meaning of participants’ experiences alongside our own interpretations [41]. The data were coded using an inductive approach. Researcher subjectivity is central to reflexive thematic analysis, which is typically led by a single coder [42]. This analysis was led by the first author, who has training in the fields of human-computer interaction and music psychology as well as experience conducting research with people living with dementia and their caregivers.

After familiarization with the transcripts, the first author generated initial codes and developed preliminary themes, which included 6 themes grouped under the category of benefits of music and technology and 5 themes grouped under the category of barriers to use. Initial themes were discussed among a subset of the authors with expertise in human-computer interaction. Initial themes were subsequently defined and named and further refined, using an iterative approach when outlining themes during the writing process. During this phase, several themes were removed, combined, or restructured based on discussions between authors and comments on draft iterations. When a full draft was complete, further discussion with the remaining authors (with expertise in music therapy), and reflection on the presentation of findings led to additional revisions. Discussions and comments led to further narrowing the focus and framing the findings around music technologies, acknowledging that music use is almost always facilitated by technology. These revisions led to the finalization of the themes presented here.

## Results

### Survey

#### Overview

The survey was launched on the web in December 2020 and was open during the first quarter of 2021. There were 77 completed surveys (people living with dementia: n=13, 17%; caregivers: n=64, 83%). Of the 13 people living with dementia, 9 (69%) consented to be contacted for the focus groups, while 24 (38%) of the 64 caregivers gave their consent to be contacted for the focus groups.

#### Survey Respondents’ Characteristics

Table 1 outlines the survey respondents’ characteristics.

In total, 13 people living with dementia responded to the survey and, where applicable, responded on behalf of their caregiver (n=8, 62% participants). Respondents living with dementia were aged between 49 and 78 (mean 65.3, SD 9.0) years and were predominantly female (9/13, 69%). Of the 13 people living with dementia, 8 (62%) responded that they had someone providing care for them. Respondents living with dementia reported that their caregivers ranged in age from 36 to 77 (mean 55.1, SD 15.1) years and were predominantly female (5/8, 63%). Most of the caregivers were a partner or a spouse (6/8, 75%). People living with dementia were primarily diagnosed with either Alzheimer disease (3/11, 27%) or frontotemporal dementia (3/11, 27%). The remainder lived with mixed dementia (2/11, 18%), another type not specified (2/11, 18%), or vascular dementia (1/11, 9%).

In addition, 64 caregivers responded to the survey and, where applicable, completed survey details on behalf of the person with dementia for whom they were caring. The caregivers ranged in age from 29 to 86 (mean 63.0, SD 11.2) years, and the majority (46/64, 72%) were female. They reported that the people they cared for ranged in age from 45 to 93 (mean 78.1, SD 9.9) years and that more than half were female (36/64, 56%). Almost half of the caregivers (30/63, 48%) were a spouse or a partner of someone living with dementia, while a little more than a third (22/63, 35%) were adult children caring for a parent with dementia.

**Table 1:** Survey respondents’ sociodemographic characteristics. Persons living with dementia answered questions about themselves. They were also asked questions about their caregiver for them to answer. Caregivers of a person living with dementia answered questions about themselves. They also answered questions about the person living with dementia for whom they were caring (n=77)^a^.

		Persons living with dementia	Caregivers	Total
Persons with dementia: age (y), mean (SD)		65.3 (9.0)	78.1 (9.9)	76.0 (10.8)
**Persons with dementia: gender, n/N (%)**				
	Female	9/13 (69)	36/64 (56)	45/77 (58)
	Male	4/13 (31)	28/64 (44)	32/77 (42)
Dementia diagnosed, n/N (%)		11/13 (84.6)	56/64 (88)	67/77 (87)
**Dementia type, n/N (%)**				
	Alzheimer disease	3/11 (27)	32/56 (57)	35/67 (52)
	Vascular dementia	1/11 (9)	5/56 (10)	6/67 (9)
	Frontotemporal dementia	3/11 (27)	5/56 (10)	8/67 (12)
	Mixed dementia	2/11 (18)	2/56 (4)	4/67 (6)
	Other	2/11 (18)	12/56 (214)	14/67 (21)
Time since diagnosis (years), mean (SD)		4.2 (2.1)	4.2 (2.9)	4.2 (2.7)
Persons with dementia with at least 1 caregiver, n/N (%)		8/13 (62)	64/64 (100)	72/77 (94)
Persons with dementia with >1 caregiver, n/N (%)		3/8 (38)	31/64 (55)	34/72 (47)
**Dyad relationship, n/N (%)**				
	Spouse or partner	6/8 (75)	30/63 (48)	36/71 (51)
	Parent and child	1/8 (12)	23/63 (37)	24/71 (34)
	Other relative or friend	0/8 (0)	3/63 (5)	3/71 (4)
	Paid carer	1/8 (12)	7/63 (11)	8/71 (11)
Caregivers: age (years), mean (SD)		55.1 (15.1)	63.0 (11.2)	62.1 (11.9)
**Caregivers: gender, n/N (%)**				
	Female	5/8 (62)	46/64 (72)	51/72 (71)
	Male	3/8 (38)	18/64 (29)	21/72 (29)

^a^Total N values differ between questions due to missing data and differences in questions displayed resulting from the participants’ responses (eg, if a person living with dementia did not respond for their caregiver).

#### Survey Respondents’ Use of Music and Technology

With regard to music use, 55% (42/76) of the people living with dementia (including those whose caregiver responded) and 75% (47/63) of the caregivers used music *often* or *very often* in their daily lives (Table 2). Furthermore, the majority of the participants (people living with dementia: 39/74, 53%; caregivers: 38/63, 60%) felt that music was *definitely* an important or meaningful part of their lives in the past. The respondents accessed music via streaming services (people living with dementia: 15/65, 23%; caregivers: 20/57, 35%), CDs (people living with dementia: 23/65, 35%; caregivers: 27/57, 47%), and radio (people living with dementia: 21/65, 32%; caregivers: 23/57, 40%). When asked about dementia-specific apps, only 15% (11/75) of all respondents had previously used one, and 62% (47/76) were willing to use a music app to support their daily activities.

When asked “What ways, if any, do you think a music-based mobile app might be able to support you in your daily activities?” people living with dementia reported that an app could help with supporting calming and relaxation (5/12, 42%) and providing entertainment or enrichment (3/12, 25%), as well as a combination of these. Caregivers responded with similar benefits, with calming and relaxation (11/57, 19%) and entertainment and enrichment (7/57, 12%) as common responses; however, 25% (14/57) of the caregivers were not certain about the benefits and suggested that a new app would have to be distinct from other music technologies to be useful to support daily activities.

**Table 2:** Survey respondents’ use of music and technology (n=77).

		Persons living with dementia, n/N (%)	Caregivers, n/N (%)	
		Respondents answering for themselves	Respondents answering for the person they care for	Respondents answering for themselves
**Current use of music**				
	Never	1/13 (8)	8/63 (13)	4/63 (6)
	Rarely	0/13 (0)	10/63 (16)	7/63 (11)
	Sometimes	3/13 (23)	12/63 (19)	5/63 (8)
	Often	3/13 (23)	12/63 (19)	19/63 (30)
	Very often	6/13 (46)	21/63 (33)	28/63 (44)
**Music was important in the past**				
	Not at all	2/13 (15)	4/61 (7)	5/63 (8)
	Somewhat	4/13 (31)	20/61 (33)	20/63 (32)
	Definitely	7/13 (54)	32/61 (52)	38/63 (60)
	Don’t know	0/13 (0)	5/61 (8)	0/63 (0)
**How they currently listen to music**				
	CDs	2/12 (17)	21/53 (40)	27/57 (47)
	Radio	5/12 (42)	16/53 (30)	23/57 (40)
	Music streaming services (eg, Spotify)	5/12 (42)	10/53 (19)	20/57 (35)
	Video streaming services (eg, YouTube)	5/12 (42)	6/53 (11)	19/57 (33)
	Digital music library (eg, iTunes library)	1/12 (8)	9/53 (17)	13/57 (23)
	Television	2/12 (17)	4/53 (8)	4/57 (7)
	Wireless speaker system	0/12 (0)	2/53 (4)	4/57 (7)
	Vinyl records	1/12 (8)	4/53 (8)	2/57 (4)
	Cassettes	1/12 (8)	2/53 (4)	0/57 (0)
	Sound therapy app	1/12 (8)	0/53 (0)	0/57 (0)
	Dementia-specific music player	0/12 (0)	1/53 (2)	0/57 (0)
Own a smartphone currently		11/13 (85)	—^a^	60/64 (94)
Own a tablet device currently		9/13 (69)	—	47/64 (73)
Experienced in the use of smartphone apps^b^		7/12 (58)	—	52/64 (81)
Experienced in the use of music apps^b^		5/12 (42)	—	36/64 (56)
Experienced in the use of dementia apps^b^		2/12 (17)	—	9/63 (14)
Experienced in the use of sound devices^b^		6/12 (50)	—	32/62 (52)
“Very” open to using a mobile app (or a new app) as part of daily activities^c^		8/13 (62)	—	39/63 (62)
**Ways in which a music-based smartphone app might be able to support daily activities**				
	For calming and relaxation	5/12 (42)	—	11/57 (19)
	For entertainment, enrichment, and reminiscence	3/12 (25)	—	7/57 (12)
	To improve or maintain mood	0/12 (0)	—	3/57 (5)
	Multiple uses	1/12 (8)	—	2/57 (4)
	General benefits	0/12 (0)	—	6/57 (11)
	New apps must be different from other music streaming services to be useful	1/12 (8)	—	14/57 (25)
	Not sure or none	2/12 (17)	—	14/57 (25)

^a^Not applicable.

^b^The answer options for this question were *no experience*, *some experience*, *quite experienced*, *very experienced*, and *expert user*; for this table, we considered *experienced users* to be those who reported the last 3 options.

^c^Respondents answered the question “How open would you be to using a mobile app (or new app) as part of your daily activities?” with *not open to the idea at all, a little open to the idea, very open to the idea,* or *undecided*.

### Focus Groups

#### Focus Group Participants’ Characteristics

Of the survey respondents willing to participate further in the study, 18 took part in the focus groups. Two people living with dementia and 2 caregivers also invited their respective care partner to participate in the focus group sessions. The focus group participants included 9 caregivers of people with dementia (partners or spouses: n=5, 56%; adult children: n=4, 44%), 3 dyads (people living with dementia: n=3, 50%; caregivers: n=3, 50% [participating together]), and 3 people currently living with dementia who attended independently. Participants ranged in age from 50 to 86 (people living with dementia: mean 72.5, SD 3.5; caregivers: mean 70.3, SD 12.1) years. Of the 18 participants, 13 (72%) identified as female, and 5 (28%) identified as male. Table 3 provides a summary of the participants’ characteristics.

**Table 3:** Focus group participants’ characteristics.

Group and pseudonym			Role		Relationship to person with dementia		Dementia type		Music technologies discussed
**Group 1**									
	Anita^a^	Caregiver		Partner		Alzheimer disease		CDs, music streaming services (with dedicated music device), vinyl records, and iPad (with music from CDs)	
**Group 2**									
	Betty	Caregiver		Child		Memory problems		Radio and music streaming services (on smartphone)	
	Caroline	Caregiver		Partner		Mixed		CDs	
	David	Caregiver		Partner		Alzheimer disease		CDs	
**Group 3**									
	Elizabeth	Caregiver		Child		Alzheimer disease		MP3 player	
	Fred	Caregiver		Partner		Alzheimer disease		Music streaming services (on smartphone and tablet device) and wireless speaker system	
	Geraldine	Caregiver		Partner of Grace		Primary progressive aphasia		Wireless speaker system, MP3 player, video streaming services, digital music library, and music streaming services	
	Grace	Person with dementia		Partner of Geraldine		Primary progressive aphasia		—^b^	
**Group 4**									
	Harriet	Caregiver		Child		Alzheimer disease		Vinyl records and music streaming services (on smartphone)	
	Ingrid^c^	Caregiver		Child		Alzheimer disease		Radio, CDs, cassettes, and music streaming services (on smartphone and tablet device connected to a Bluetooth speaker)	
	Ian^c^	Caregiver		Partner		Alzheimer disease		Radio and CDs	
**Group 5**									
	James	Caregiver		Partner of Julia		Alzheimer disease		CDs, radio, music streaming services (on smartphone), video streaming services, and piano apps	
	Julia	Person with dementia		Partner of James		Alzheimer disease		—	
	Karen	Caregiver		Partner of Kevin		Lewy body		—	
	Kevin	Person with dementia		Partner of Karen		Lewy body		Radio and music streaming services	
	Lucy	Person with dementia		Attended independently		Mixed		CDs and MP3 player	
	Mary	Person with dementia		Attended independently		Mixed		Radio and CDs	
	Naomi	Person with dementia		Attended independently		Frontotemporal		Radio and vinyl records	

^a^Focus group 1 was conducted with 1 participant because 2 others who were scheduled to join withdrew due to unforeseen circumstances.

^b^Not applicable.

^c^Caregivers Ian and Ingrid are father and daughter, both caring for the same person—Ian’s partner and Ingrid’s mother—who did not participate in the study.

#### Benefits of Music Technologies: How Caregivers and People Living With Dementia Use Music as Part of Their Everyday Care Practices

Caregivers and people living with dementia described four benefits of using music technologies in everyday care: (1) to regulate mood and provide joy, (2) to facilitate music-based social activities, (3) to provide continuity of music use before and after a clinical diagnosis, and (4) to make caregiving easier.

##### Using Music for Regulating Mood and Providing Joy

Caregivers said that they often used music and music technologies as a tool for regulating the mood of their loved ones in their everyday lives and in care practices. Caregivers believed that music was valuable for its ability to help manage the behavioral and psychological symptoms of dementia and provided joy for both caregivers and people living with dementia. Caregivers chose to listen to music out loud through shared technologies (eg, speakers) because they perceived these potential benefits for their loved ones; for example, Harriet (all names used in the quotes herein are pseudonyms) found that listening to vinyl records could be valuable in managing her mother’s mood:

I would say [music] lifts her mood...she does get a bit down...gets a bit paranoid sometimes, but I just think music has a calming effect and it does definitely lift her mood.

For Fred and his wife, music had a relaxing effect even when “choosing different playlists [that are] not specific for calming.”

Ingrid, who cared for her mother during the day, noted the benefits of music listening as part of a routine and at difficult times of day, notably “sundowning”:

And if the light is changing, and I know that there’s still two to three hours before Papa arrives to collect her...I say: “Shall I put some music on?” And she said: “Oh yes, please.” And I find some classical works extremely well, and anything that’s calming...gentle jazz.

As well as specific emotional impacts of music, caregivers and people with dementia discussed the benefits of using music to manage the behavioral symptoms of dementia; for example, Ian, also speaking about Ingrid’s mother (his partner), stated as follows:

[She is] a bit prickly at the moment...and then occasionally she will lash out at those closest to her, so I use music as a pacifier.

In terms of the technologies used to access music, their portability provided benefits such as allowing music to be used when and where it was needed as part of emergent care practices for both the person living with dementia and their caregivers. Lucy, a person living with dementia, said that she found it useful to keep a small MP3 player close by to help her manage a particularly troubling symptom of dementia: hallucinations that occurred at night. Lucy found that using her MP3 player to listen to music helped her calm down during these hallucinations.

Furthermore, the portability of some music technologies such as MP3 players or streaming services such as Spotify has made music an accessible means of emotion regulation outside of the home. Anita, caring for her husband, discussed the benefits of streaming music from a mobile device:

It’s also then something that I can take with us, you know...there are occasions when we have to go to appointments and things like that and if there is waiting time then you know I can use it there...if I feel him getting a bit agitated or anything.

While music listening offers a range of practical benefits for people with dementia and their caregivers, participants also noted the fun and enjoyment that comes from actively engaging in music activities. Active forms of engagement such as singing, playing instruments, and moving to music were discussed. In multiple examples, participants with dementia and caregivers spoke of the joy they experienced when singing along to music; for example, Naomi, a person living with dementia, stated as follows:

Now because I’m having struggle speaking, but I have the joy of being able to sing...it’s so much easier to sing now than actually talk some days.

Other enjoyable activities included moving and dancing to music. Geraldine, a caregiver, spoke of how she and her partner could enjoy music and dancing together by streaming music through wireless speakers in their home:

We have a playlist that we both like and every now and then...one of us might go to the other person’s room wherever they’re cleaning you know and have a dance because it’s...particularly memorable for us.

These examples suggest that music activities beyond listening can offer benefits for people with dementia and their caregivers. Participants described how every day and spontaneous activities, such as singing and dancing to music, can be supported using technologies that are available within the home (eg, wireless speakers).

##### Technologies Can Facilitate Music-Based Social Activities in Care

Music and music technologies were reported to encourage social interaction. Participants shared examples that showed how participating in organized music activities facilitated social bonding for those still living at home. These activities were supported by technology that provided access to music resources beyond the setting of the organized activity.

In the home care context, Geraldine, a caregiver, described attending a social musical activity for people with dementia and their caregivers:

[W]e had another session which was a karaoke sing along for people and it was...through a care group so it wasn’t necessarily all for people with dementia, but it was for people caring for other people...but any kind of interaction I think brings so much more to a person’s life, if they are involved in doing something while the music is playing.

These music activities were sometimes extended with technologies used in the home; for example, James, who cared for his wife at home, shared how they used a music app to assist with singing in their local choir:

I use certain apps to do the choir sections...when you sing in a choir, it’s very hard to get your pitch right. So, you use your app, that’s why I’ve got a keyboard to try and play the parts of the choir. So, you download, basically using YouTube and the facilities there they give you choir sections which break up the score into alto, soprano and so on.

In other words, James and his wife used resources available through YouTube to practice their parts for the choir.

Music technologies were important because they not only enabled participants to share music with others—for example, by listening to music together—but also made it possible to experience music and a sense of companionship independently. Ingrid, who cares for her mother with dementia and also hosts a radio show, highlighted the role that music technologies, such as radio, can play in fulfilling social needs:

So, radio again...it’s very personal...literally there is somebody in the room there and you don’t have to recognize them. You can recognize the voice, for example, you can hear, say, certain voices are absolutely unmistakable when you hear them on radio.

##### Music Technologies Provide Continuity of Music Use Before and After the Diagnosis

One of the key benefits caregivers shared was that music technologies enabled continuity in music listening throughout the different stages of their experience with dementia. Caregivers and people living with dementia described their existing relationships with music and explained that listening to music provided a link between experiences before and after the dementia diagnosis.

Participants living with dementia primarily preferred listening to older styles of music from their youth; yet, caregivers also noted the potential benefits of other styles. Music from earlier decades was often familiar, and despite experiencing difficulties with memory, people with dementia still remembered songs from their youth and enjoyed singing along to them, as noted by Kevin, who has Lewy body dementia:

With the Lewy body, it doesn’t affect your long-term memory really, it’s the short-term memory. So, a lot of the old songs, you can remember. It just comes out and you sing.

While there was substantial emphasis on jazz and classical music as genres people with dementia “used to like listening to” (Caroline), participants also noted that music taste was not fixed and that other genres could provide benefits, as noted by Elizabeth, who cared for her mother:

I had this...idea that, you know, mum loves Ella Fitzgerald and all those old female singers and Frank Sinatra...and I sort of had this, right it’s gotta be all those oldies or Peter Allen ‘cause she loves him and those songs from the ’70s. But I had some success with...like one of my kids would play Taylor Swift or something...and that sort of calmed her.

Music collections were predominantly gathered in the years before the dementia diagnosis, and music from the individuals’ formative years was prominent. The technologies used were diverse across participants, often reflecting participants’ music use before their diagnosis; for example, Harriet noted that her mother had “a big record collection,” while Anita’s husband had “CDs from...singers he would have grown up listening to.” Radio was popular with both people living with dementia and caregivers, particularly stations that played classical music; for example, Lucy shared that radio “kept [her] sane” during the COVID-19 pandemic, while caregivers, such as James and Ingrid, discussed turning on the radio regularly because it was a familiar technology, as Ingrid explained:

So music in radio is very, very powerful because that’s also a medium, in actual fact it was the medium, apart from cinema, for my parents’ generation growing up.

In addition, Fred continued to use music streaming services, with “playlists that [were] put together some years ago before the dementia set in.”

Notably, despite the positive effects of music listening, participants also recalled negative experiences. In particular, music could be associated with memories that were not always welcome for the person living with dementia. After trialing an MP3 player and headphones with her mother, Elizabeth noted as follows:

For my mum it [music] triggered a lot of memories and she wanted to shut them down, and music upset her very much.

For many caregivers, playing music was used as an important way to facilitate reminiscence and, as Ian described, “a different form of bringing back reality” for their loved ones; for example, Ingrid (speaking about her mother, Ian’s partner) had a sense that music was special for her mother, sharing that they often played the radio for her:

Sometimes she doesn’t recognize me but recognizes my voice. So, I do a jazz program on community radio every 3-4 weeks...and dad...will have the radio on and she will recognize my voice, and...[she] will recognize the music and the pieces, particularly if they are ones that I was familiar with growing up...So...there will be like a little trigger there.

Participants highlighted how streaming services and MP3 players can be used to create tailored playlists that foster reminiscence for people living with dementia, but these playlists need to be used carefully, given that music can also provoke painful memories, as highlighted by Elizabeth. While the link between music and reminiscence is well known, these examples show that family caregivers are informally using music technologies to connect with their loved ones, including being mindful of both the continuity and the positive link with past experiences that music can evoke and the risk of triggering difficult memories when listening to music.

##### Music Technologies Help Make Caregiving Easier

As well as using music to directly support their loved ones, caregivers also used music technologies to address their own needs. By providing entertainment and relaxation for the person they were caring for, music freed up time for the caregiver and helped to make caring easier for participants and others providing care for their loved ones. In both situations, new music technologies made it easier for caregivers to use music strategically in their caregiving.

When used to provide an enjoyable distraction for the person living with dementia, music freed up caregivers’ time to attend to other activities. Participants explained that newer technologies (eg, streaming services) had advantages over older modalities (eg, vinyl records) in this regard, because they could be left to play for longer, as Anita explained:

We have lots of old records...but I find that if I play the music there, I’ve no sooner got it on than it seems to stop...[it] interrupts another activity that I might be involved in so I find the newer technologies leave me freer to do other things here at home for a longer time.

Music was used not only by the primary caregiver but also by others in the care network. Fred had set up music technologies in the home before his wife’s diagnosis. This allowed visiting caregivers to use these technologies to play music when caring for Fred’s wife:

There’s a carer in each day...and if I’m out they can still tap into [the Wi-Fi] and play music that’s on their [phone]...That’s another big win now, exploring with that so they can get music going for [his wife] and either their own music, their own playlists, individual songs, etcetera.

Finally, caregivers described using music technology to entertain their loved one. David mentioned putting on music in the background so that his wife was not sitting in silence. Anita said that she frequently plays CDs at home to fill spare time:

I just use it in those times, when there’s really nothing else [to do] apart from sleep and just sitting staring.

#### Barriers for Music Technology in Care

Despite the potential advantages of using music-listening technologies in care, several issues with technology use were raised by the participants. Barriers to use centered around a difficulty for caregivers and people living with dementia to stay up to date with constantly evolving technologies as well as issues with confidence and ability to use digital technologies.

##### Difficulty Staying Up to Date With Evolving Technology

Participants described difficulties staying up to date with evolving technology in terms of smartphone apps and other music technologies. Even if familiar, these technologies sometimes became more difficult for people living with dementia and caregivers to use over time.

As a self-described older caregiver, James said he experienced difficulties with the planned obsolescence common to new digital technologies:

To try to adapt as you’re older to the phones, and they’re designed to be out of date within six years and you gotta find a new one and it all changes, I just find it...very difficult to negotiate.

Caregivers faced similar barriers with smartphone apps. Geraldine mentioned an instance when she and her partner with dementia stopped using an app that they had previously found useful. The app was practical until they changed from one smartphone brand to another where the app displayed differently, and they encountered errors:

[Grace] had an iPhone at the time and then changed over to Samsung, and it was an Apple app. The Samsung version is not nearly as user friendly...the Apple one was just visually clearer...seemed to be easier to upload [photos]...this one...got stuck...it wouldn’t let me [upload]...So Grace doesn’t use that so much now.

When discussing other music technologies, Harriet said her mother still relies on vinyl records, while David, caring for his wife, stated that he still uses CDs and does not know how to use newer forms of music technology:

I would prefer CDs I think because they’re controllable...and there are different ones. Presumably you can do that all digitally but I don’t really know how to do that.

Similarly, Lucy, a person living with dementia, often listened to the radio but believed that other digital technologies might make music listening easier if she could learn how to use them. Lucy did use an MP3 player but faced difficulties downloading music onto the device:

It’s like, I know music, listening to it in those little...iPods...because it’s handy in the night if I feel like the traumas are coming up or some hallucinations. Immediately put that [on], it will at least calm me down. But I don’t have the know-how, how to do that...the downloading...I’m not very good at.

##### Participants With Dementia May Have Low Self-Efficacy With Technology

While older technologies tended to be more familiar, people with dementia still faced challenges with using some technologies and expressed low self-efficacy with technology; for example, Harriet found that her mother often had trouble putting on a vinyl record:

I think she panics. She panics with anything with any buttons. If she calms down enough, she’ll do it. But if she starts overthinking it, then she just panics and can’t do it.

Mary, a person living with dementia, stated that it was common for people with dementia to feel overwhelmed when talking about smartphone technologies:

As you talk “app,” nine-tenths of the people I know with dementia are going to back away because that’s beyond them.

As a result of these challenges, several caregivers discussed how important it was that apps and smartphone-based technologies were easy to use, as explained by Harriet:

I’m just all about functionality: easiness. Basically, the easier the better. The more basic, the better.

The accessibility of smartphone technology was also considered at length, with participants discussing hearing issues (Betty) and vision impairment; for example, James said that “[a] bigger screen is definitely helpful rather than small screens.” There were also physical issues with using mobile technology, as noted by Ingrid:

[I]f I’m typing [on the phone]...I really have to be very careful, how I type, and...that’s just me [a younger person] so for somebody who no longer has agility, let alone mobility with fingers, that could be an issue.

In addition, some participants perceived smartphone apps as difficult to use for people living with dementia; for example, Betty was concerned that setting up a music app for her mother with dementia would require “knowledge, information or training for the person setting it up, which probably may well be the carer.”

## Discussion

### Principal Findings

Using a mixed methods design, this study aimed to understand how people living with dementia and their family caregivers use music and music technologies in their everyday caring, as well as the challenges they experience using music technologies. In our survey, we found that the majority of participants living with dementia (9/13, 69%) and caregivers (47/63, 75%) use music *often* or *very often* in their daily lives, with music having been an important or meaningful part of their lives in the past. Consistent with prior work, participants reported using a range of methods and devices for accessing music, with high rates of use for music and video streaming services, as well as the use of more traditional music technologies such as CDs and radio [43]. Participants were interested in new ways of using music to support their care, with a willingness to try new music-based mobile apps, and considered a range of potential benefits such an app could provide. These findings, alongside previous work supporting the important role that music can play in the everyday lives of people living with dementia [24], suggest that new music technologies can be designed to complement and extend existing uses. New technology-based solutions, if designed appropriately to ensure ease of use and adding no further burden to caregivers, may provide valuable benefits for this group.

Through the focus groups, we found several perceived benefits of using music technologies, alongside the challenges that caregivers and people living with dementia face in using music and technology in these contexts. We found that people living with dementia and their caregivers use music for regulating mood, providing joy, and encouraging social connection, as well as to make caregiving easier, provide continuity, and promote reminiscence. These uses align with benefits identified in research evaluating music interventions with people living with dementia [11,23,25,28,31]; however, we additionally highlight how these benefits are demonstrated in everyday caregiving practices. We also identified barriers to using music technologies, predominantly due to participants with dementia having low self-efficacy with technology and challenges for both caregivers and people living with dementia in staying up to date with evolving technology. Here, we consider how future music technologies can be designed to capitalize on the existing uses of music technology among caregivers of people living with dementia and to empower caregivers to use music strategically and therapeutically at home [7].

People living with dementia and caregivers predominantly used music during everyday care for mood regulation, such as relaxation, to lift mood, and to provide joy. This extends prior research findings that caregivers and people living with dementia often use music for specific functions, such as relaxation or calming [23]; for example, Ingrid listened to music with her mother to manage behaviors such as late-day confusion and restlessness. In addition, participants spoke fondly of music’s ability to simply provide joy, echoing previous work where music and sound technologies have been shown to enrich the lives of people living with dementia and provide enjoyable moments at home [29]. This highlights the need for music technologies and interventions to consider the enjoyable and recreational aspects of music as well as its potential therapeutic benefits [11]. Importantly, digital technologies provide an avenue for these benefits to be translated to situations outside of the home, with Anita and Lucy suggesting that music could be a convenient and portable mood regulation tool.

The role of music and music technologies for supporting social connection, both within a care dyad and with others, was evidenced in our study and aligns with prior research [25,29,31-33]. Participating in music activities outside of the home fostered social connections for people living with dementia, as did music activities within residential aged care settings. Extending prior work, our findings show how everyday technologies were important to these interactions; for example, James discussed how mobile apps supported him and his partner in practicing their choir singing.

We found that caregivers and people living with dementia described existing relationships with music, outlining some continuity between experiences before and after the dementia diagnosis. Music technologies facilitated this continuity, enabling caregivers to encourage the use of familiar technologies, such as CDs and vinyl records, as well as personalized playlists. Music use was often associated with reminiscence, with people living with dementia reporting that they could sing along to old favorites, even if they experienced challenges with memory or other forms of verbal communication. Consistent with the idea of the “reminiscence bump,” where individuals tend to favor the music of their youth [44-46], many people living with dementia preferred older styles of music. Existing relationships with music were also connected with collections of vinyl records, CDs, and favorite radio stations, with our findings also illustrating the importance of radio for older adults during everyday care [47,48].

Finally, consistent with previous work [25,49], music technologies were important for making caregiving easier, such as accessing music to fill time or silence. We extend these findings, showing that modern music technologies, such as wireless speakers, enabled music to be played for the person living with dementia not only by the primary carer but also by others in the broader care network, such as visiting caregivers, with minimal setup. This enhanced everyday care for our participants.

### Barriers and Design Opportunities

While previous research has identified the uses of music in residential aged care settings [13,23] and designed tools for music listening, sound, and music activities in dementia care [27-30,33,34], there has been limited research investigating the everyday uses of music technologies in care practices by family caregivers of people living with dementia. To date, no research has clearly identified opportunities for music technologies to play a role in the therapeutic use of music at home. Building on our findings, ensuring the ease of use of music technologies is likely crucial for engaging caregivers and people living with dementia in music use, both for its therapeutic benefits and for recreational uses of music. This is especially pertinent given that the major barriers to music technology use in everyday care included difficulties with self-efficacy around technology use and challenges staying up to date with evolving technology. Previous research has established the importance of digital literacy and self-efficacy in technology use among older adults [50], indicating that this is an important consideration when designing technologies for people living with dementia. Therefore, if caregivers and people living with dementia are not confident using existing technologies to support music use, co-designing new technologies that capitalize on the everyday ways in which these groups use music technologies should be a priority.

Our findings suggest several design opportunities for future technologies. New technologies should be designed to align with how music is used to regulate mood and capitalize on the ways in which music technologies can be used as a portable emotion regulation tool. Music technologies can also be used to foster social interaction between people living with dementia and their caregivers, with videoconferencing tools and apps designed to support web-based music activities, such as choirs and dancing. In addition, in designing music technologies for people living with dementia and their caregivers, access to personally preferred music is crucial. Often, this may be from the reminiscence bump (ie, music from a person’s youth); however, music taste should not be assumed nor limited to older styles. These considerations for music selection and the affordances of personalized playlist creation highlight the benefits that streaming platforms might have over physical music collections, as long as they are easy to access for both people living with dementia and caregivers. One way this could be achieved is through playlists configured to play on a smart speaker with an easy-to-remember prompt that activates the desired playlist. Research has shown that voice interaction devices such as smart speakers (eg, Amazon Alexa and Google Home) have accessibility advantages over screen-based technologies for some users [51-54]. Music technologies can also be designed to make it easy for caregivers to play recorded music and use music in intentional ways to relieve pressure in family caregivers [6]. This could extend to co-designing new music technologies that train caregivers to use music therapeutically in their everyday care [7,55]. Focusing on such factors and ensuring that people living with dementia and their caregivers are comfortable using such technologies is a crucial first step to supporting the use of music technologies in care.

### Strengths, Limitations, and Future Directions

This is the first study to our knowledge that provides an in-depth understanding of how caregivers and people living with dementia use music and music technologies in their everyday caregiving practices, outside the context of a formal intervention. Understanding these everyday uses of music technologies in caregiving highlights opportunities for new technological innovation. We intend to use these findings to inform the design of future music-based technologies for this setting, capitalizing on these existing uses of music technologies. A further strength of this study was the involvement of both caregivers and people living with dementia in our survey and focus groups, recognizing the importance of understanding the perspectives of both groups.

One limitation of this study was that due to the COVID-19 pandemic at the time of recruitment, the complexity of this care setting, and the aim to recruit participants across Australia, recruitment and data collection took place entirely on the web. As such, our sample may not be representative of people living with dementia and caregivers with less technology experience. Future research may consider recruiting participants with a more diverse experience with digital technologies. Furthermore, our findings may not be generalizable outside of the Australian context.

### Conclusions

Informal caregivers of people with dementia report that they need additional support and information [4]; yet, they are often reluctant, or lack knowledge, to fully use services [5]; in addition, many family caregivers experience high levels of stress and burden [6]. As a result, caregivers and people living with dementia need accessible, everyday strategies to draw on to support their needs, and technologically supported music use is a key avenue for future research [7,9,27]. This study draws attention to the ways in which these groups already use music technologies to support their needs, outside the context of organized activities or targeted music interventions. Caregivers and people living with dementia believe that music technologies may be a useful way to access music for regulating mood, providing joy, encouraging social connection, providing continuity, promoting reminiscence, and making caregiving easier. The technologies used to play music are diverse across users, and people living with dementia and their caregivers face barriers to using new digital music technologies, despite their acknowledged advantages over more familiar technologies. These findings will contribute to understanding how new music technologies and interventions may complement existing practices. There is a clear opportunity to design technologies to capitalize on existing music use in these groups to enable easier access to specific music activities to support people living with dementia with recreational and therapeutic music listening as well as music-based activities.

## Appendix

Multimedia Appendix 1 Survey questions and semistructured interview guide.

## Abbreviations

REDCap: Research Electronic Data Capture
