# Author Correction: Everyday Uses of Music Listening and Music Technologies by Caregivers and People With Dementia: Survey and Focus Group Study

**DOI:** 10.2196/65977

**Published:** 2024-09-09

**Authors:** Dianna Vidas, Romina Carrasco, Ryan M Kelly, Jenny Waycott, Jeanette Tamplin, Kate McMahon, Libby M Flynn, Phoebe A Stretton-Smith, Tanara Vieira Sousa, Felicity A Baker

**Affiliations:** 1 School of Computing and Information Systems The University of Melbourne Parkville Australia; 2 Facultad de Comunicación y Artes Audiovisuales University of Las Américas Ecuador Quito Ecuador; 3 School of Computing Technologies RMIT University Melbourne Australia; 4 Faculty of Fine Arts and Music The University of Melbourne Melbourne Australia; 5 Centre for Research in Music and Health Norwegian Academy of Music Oslo Norway

In “Everyday Uses of Music Listening and Music Technologies by Caregivers and People With Dementia: Survey and Focus Group Study” (J Med Internet Res 2024;26:e54186) the authors noted one error.

The ninth author's name was listed as:

Tanara Viera Sousa

This has been changed to the following:

Tanara Vieira Sousa

The correction will appear in the online version of the paper on the JMIR Publications website on September 9, 2024, together with the publication of this correction notice. Because this was made after submission to PubMed, PubMed Central, and other full-text repositories, the corrected article has also been resubmitted to those repositories.

