# Supplementary material for: Everyday Uses of Music Listening and Music Technologies by Caregivers and People With Dementia: Survey and Focus Group Study
Source: J Med Internet Res. 2024 Aug 27;26:e54186. doi: 10.2196/54186 (PMC11387911; doi:10.2196/54186)
Supplement: Multimedia Appendix 1 [file jmir_v26i1e54186_app1.docx]

**Appendix A:**

| This survey aims to collect data about family caregivers and people living with dementia’s use of music and technology which will inform the development of a specialised mobile app designed to support the daily care of people living with dementia at home.  Music therapy is an allied health discipline that has strong evidence for supporting the wellbeing of people living with dementia and their family caregivers. As the incidence of dementia increases worldwide, individualized face-to-face therapy programs are increasingly unfeasible. A technology-based program could help to ensure the benefits of music therapy becomes more widely available to family caregivers and people living with dementia, not just to those who are working directly with a music therapist.  We therefore need to explore how technology can support therapy-informed music use in dementia care. We hope to use the information we get from this research to inform the development of a mobile App that is designed to support family caregivers in the use of music in the home that will enhance their experience of caring.  If you agree to participate in this study, you will be asked at the beginning of the project to complete a short anonymous online survey to provide basic demographic information (such as age, sex, level of education achieved and previous experience with music and technology). The survey consists of 36 questions and is anticipated to take approximately 15-20 minutes to complete. As a thank-you for your participation, we will offer you an opportunity to go into the draw to win an iPad MINI.  Participation in this study will not give you any direct benefit; however, your perspectives will contribute to the design of an app that will be useful for supporting other carers such as yourself. |
| --- |
| Are you a person living with Dementia or a caregiver?   \| **YES,  I am a person living with Dementia** \| **YES,  I am a caregiver of a person living with Dementia** \| **NO**  *Thank you for your interest in our project. If you want to know more about HOMESIDE Projects, please follow us here:* [*www.homesidestudy.eu/*](http://www.homesidestudy.eu/)  *If you know a person living with Dementia and/or Caregivers, please share this link.* \| \| --- \| --- \| --- \| |

| **Firstly...** |
| --- |
| If you are a person living with Dementia, you will answer questions about yourself. We will use the words “Care Recipient” to refer to questions about you. If you have a member of your family or a friend who is supporting you in your daily care, we may ask questions about this person for you to answer and this person will be called “Caregiver”. |
| If you are a Caregiver of a person living with Dementia, you will answer questions about yourself. We will call you in following sections "Caregiver", and you will also answer questions about the person living with dementia who you are caring for. We will refer to this person as the "Care Recipient" in the next sections |
| **We will start with information about the Care Recipient** |

| Age: ______ (in years) | Sex:   - ¨ Male - ¨ Female - ¨ Non-gender specific |
| --- | --- |
| Country of birth?   - ¨ Australia - ¨ New Zealand - ¨ Other ____________________________ | Languages:   - ¨ English - ¨ Other (s) ____________________________ |
| Highest level of education completed:   - ¨ No formal schooling - ¨ Primary school - ¨ Secondary or high school - ¨Trade, community or TAFE college - ¨ Bachelor’s degree - ¨ Masters Degree - ¨ PhD - ¨ Other_______________________________________ | |

| **This section is about the Dementia Diagnosis** |
| --- |

| Has your doctor identified a problem with short-term memory or perhaps a diagnosis of dementia?   - ¨ No - ¨ Yes, memory problems - ¨ Yes, we received a diagnosis | What type of dementia was diagnosed?   - ¨ Alzheimer’s Disease - ¨ Vascular Dementia - ¨ Lewy Body Disease - ¨ Frontotemporal Dementia - ¨ ETOH Related Dementia - ¨ Other. Please specify: __________________ |
| --- | --- |
| Approximately, how long ago did you receive the dementia diagnosis or started to present with memory problems? ______________ (in years) | |
| Do you have someone providing care for you, due to your condition? *(only for CR)*   - ¨ Yes - ¨ No (skip for **section about use of Music**) | |

| Now, answer about the relationship between Care Recipient and Caregiver |
| --- |

| What is the relationship between Caregiver and Care Recipient:   - ¨ Spouse/partner - ¨ Sibling - ¨ Child - ¨ Friend - ¨ Other _____________________________ | Is the Caregiver paid (only for non-relatives)?   - ¨ Yes - ¨ No |
| --- | --- |
| How many other caregivers are involved in your care?  ¨ 0  ¨ 1  ¨ 2  ¨ 4  ¨ 4 or more  ¨ I don’t know | |

| **Now, information about the Caregiver** |
| --- |

| Age: ______ (in years) | Sex:   - ¨ Male - ¨ Female - ¨ Non-gender specific |
| --- | --- |
| Country of birth?   - ¨ Australia - ¨ New Zealand - ¨ Other ____________________________ | Languages:   - ¨ English - ¨ Other (s) ____________________________ |
| Highest level of education completed:   - ¨ No formal schooling - ¨ Primary school - ¨ Secondary or high school - ¨Trade, community or TAFE college - ¨ Bachelor’s degree - ¨ Masters Degree - ¨ PhD - ¨ Other_______________________________________ | |

| Now, about use of Music... |
| --- |

|  | **Care Recipient** | **Caregiver**  *(let blank if you don’t have a Caregiver)* |
| --- | --- | --- |
| Do you currently use music in your daily life? | - Never - ¨ Rarely (less than once per week) - ¨ Sometimes (about once per week) - ¨ Often (several times per week but less than every day) - ¨ Very often (once or more per day) | - Never - ¨ Rarely (less than once per week) - ¨ Sometimes (about once per week) - ¨ Often (several times per week but less than every day) - ¨ Very often (once or more per day) |
| Has music been an important or meaningful part of your life in the past? | - ¨ Not at all - ¨ Somewhat - ¨ Definitely | - ¨ Not at all - ¨ Somewhat - ¨ Definitely |
| What are your musical preferences? (e.g. genres/styles, bands, singers, songs/pieces, instruments) |  |  |
| What (if any) equipment do you or the Care Recipient currently use for listening to music? E.g. Records, tapes, downloaded music, YouTube, streaming applications (e.g. Spotify, YouTube, Apple Music) |  |  |

| **About your use of technology...** | | | | | |
| --- | --- | --- | --- | --- | --- |
| **How would you describe your level of experience using the following types of technology?** | | | | | |
|  | **No Experience** | **Some Experience** | **Quite Experienced** | **Very Experienced** | **Expert User** |
| Tablet (i.e. iPad, Android, or Windows Surface) |  |  |  |  |  |
| Desktop Computer (i.e. Windows or Mac) |  |  |  |  |  |
| Smartphone (i.e. iPhone, Android, or Windows Phone) |  |  |  |  |  |
| Smartphone Apps (e.g. WhatsApp, Facebook) |  |  |  |  |  |
| Music Apps (e.g. Spotify, YouTube, Apple Music, Google Music) |  |  |  |  |  |
| Music Devices (i.e. bluetooth speakers, headphones) |  |  |  |  |  |
| Dementia related Apps (e. g. Dementia Diary, Dementia Clock ) |  |  |  |  |  |

| Do you have access to fast (broadband) internet at your place of residence?  🞎 Yes  🞎 No |
| --- |
| Do you currently own a smartphone and/or a tablet?  🞎 Yes, a smartphone. What kind (e.g. iPhone, Android, Huawei, Windows): ______________________________  🞎 Yes, a tablet. What kind (e.g. iPad, Samsung or other using Android ): _________________________________  🞎 Neither |
| How often do you use your smartphone and or tablet?  🞎 Every few minutes  🞎 A few times an hour  🞎 About once an hour  🞎 A few times a day  🞎 About once a day  🞎 Less than once a day |

| **Now, about the use of Mobile Apps specific for Music or Dementia** |
| --- |

|  | **Mobile App 1** | **Mobile App 2** | **Mobile App 3** | **Mobile App 4** |
| --- | --- | --- | --- | --- |
| Name of Mobile Apps if any, being used to help you: |  |  |  |  |
| Please briefly describe how this Mobile App helps you? |  |  |  |  |
| Are you paying for this Mobile App? | 🞎 No, it is free  🞎 No, I use the free version  🞎 Yes, I pay for the advanced version  (if available) | 🞎 No, it is free  🞎 No, I use the free version  🞎 Yes, I pay for the advanced version  (if available) | 🞎 No, it is free  🞎 No, I use the free version  🞎 Yes, I pay for the advanced version  (if available) | 🞎 No, it is free  🞎 No, I use the free version  🞎 Yes, I pay for the advanced version  (if available) |

| Would you be willing to use Mobile Apps (or a new App in case you already use some) in your daily activities as a person living with dementia?  🞎 Yes  🞎 No |
| --- |
| What do you expect from a Mobile App to help in your daily activities?  _____________________________________________________________________________________________  _____________________________________________________________________________________________ |
| If a Mobile App was available to help you in your daily activities, how would you rank the below options to best describe your willingness to access it?  _______ I would be willing to pay a periodic subscription fee (monthly, annually, etc.)  _______ I would be willing to pay a per-use fee  _______ I would not be willing to pay any fee, but have it funded by advertisement or company  _______ I would not be willing to pay any fee, but have it provided by the Government or other organisation |
| How much would you be willing to pay for a Mobile App in a monthly subscription fee format? ________ (in AUD) |
| In your opinion, where would you be most likely to hear about these Mobile Apps?  ¨ Through Dementia Organisations and Universities  ¨ Through advertisement or referral from my GP and health specialists  ¨ Through TV, Radio or Internet advertisement  ¨ Other channels ______ |
| **Now, to finalise...** |
| Would you like to comment on something else?  _____________________________________________________________________________________________  _____________________________________________________________________________________________ |

| Now, we would like to invite you to participate in the second part of the study. |
| --- |
| *Thank you for completing the survey. This next part is an invitation to participate in an online workshop about developing an app that supports the caregiver and person living with dementia to use music in a meaningful way. I encourage you to read the brief description below.*  *We will offer a store-voucher to the value of $30 as a token thank-you for your participation should you consent to participate. Clicking in the box YES below will take you to a more detailed explanation of the workshop. If you do not want to proceed click NO and you may close the webpage.* |
| Do you want to know more about this study?   \| **Yes** \| **No** \| \| --- \| --- \| \| *Continue on to the* ***Plain Statement Language*** \| *Thank you for your participation in the first stage of this project. If you want to know more about HOMESIDE Projects, please follow us here:* [*www.homesidestudy.eu/*](http://www.homesidestudy.eu/) \| |

**Appendix B:**

1. Introductions: (10 minutes)

Welcome to our first design workshop. In this workshop we would like to know if and how you currently use music and technology when caring for your loved one and how you think an app could support you in caring for them. Your comments and experiences will help us to design a mobile app that helps train caregivers to use music in a more purposeful way to help reduce or support certain symptoms of Dementia and improve quality of life from both the person living with dementia and you, the carers.

Before we start, I would like to remind you that this workshop will be recorded. If you are uncomfortable with your face appearing on the recording, now would be a good time to ensure your camera is turned off. The recording of this workshop will not be shared outside the research team.

To begin, we would like to know more about you, please tell us your name and tell us a bit about your role as a carer.

[Direct participants introduce themselves. Make sure everyone has a chance to introduce themselves]

2. Brainstorming Activity About Music Technologies at Home: (25 minutes)

In this first section, we would like to find out if and how you use music and technology when caring for your loved one.

To get us started, can you each please take a few minutes to think of times when using music has helped you care for your loved one. Can you tell us what happened?

[GROUP DISCUSSION: Participants share their stories]

Now, I will like you to describe any incidents you have experienced when the music didn't go that well.

[GROUP DISCUSSION: Participants share their stories]

Now, I will like you to think about the technologies you currently use (not just music technologies but any technologies)? What apps are you currently using? What features do you find engaging? What features stop you from using these apps? What do you wish the apps could do?

[GROUP DISCUSSION: Participants share their experiences, and a research team member will make notes with these responses on a shared screen]

Now to finish off this section, please think of these two things together: music and technology. What music technologies do you prefer and why? How do these technologies support or not support your caring activities?

[GROUP DISCUSSION: Participants share their stories, and a research team member will create a Miro board with these responses on a shared screen]

3. The music assistant: (20 minutes)

Imagine that you are going to help us create a “music assistant” app that will help you use music when caring for your loved one. What do you imagine this app could do?

You can imagine anything, remember that all ideas are welcome and that it will be okay if we do not agree with each other. Some people will like features that other people don't like and that's okay.

[GROUP DISCUSSION: Participants share their ideas, and a research team member will create a Miro board their responses on a shared screen. Make sure everyone has a chance to contribute their ideas.]

4. Retrospective Wrap-Up: (5 minutes)

We have come to the end of the workshop. We want to thank you for your time and for sharing your experiences and ideas. It has been great to hear from you. Your comments and experiences will help us to design a mobile app that better responds to the needs of carers.

As a thank you for your time, you will receive a voucher. In the following days, we will be contacting you to arrange these details.

End of the activities
